# Mechanism-Driven Modeling to Aid Non-invasive Monitoring of Cardiac Function *via* Ballistocardiography

**DOI:** 10.3389/fmedt.2022.788264

**Published:** 2022-02-16

**Authors:** Mohamed Zaid, Lorenzo Sala, Jan R. Ivey, Darla L. Tharp, Christina M. Mueller, Pamela K. Thorne, Shannon C. Kelly, Kleiton Augusto Santos Silva, Amira R. Amin, Pilar Ruiz-Lozano, Michael S. Kapiloff, Laurel Despins, Mihail Popescu, James Keller, Marjorie Skubic, Salman Ahmad, Craig A. Emter, Giovanna Guidoboni

**Affiliations:** ^1^Electrical Engineering and Computer Science, College of Engineering, University of Missouri, Columbia, MO, United States; ^2^Centre de Recherche Inria Saclay Île-De-France, Palaiseau, France; ^3^Biomedical Sciences, College of Veterinary Medicine, University of Missouri, Columbia, MO, United States; ^4^Department of Biomedical Sciences, Cooper Medical School of Rowan University, Camden, NJ, United States; ^5^REGENCOR, San Carlos, CA, United States; ^6^Departments of Ophthalmology and Medicine, Stanford Cardiovascular Institute, Stanford University, Palo Alto, CA, United States; ^7^Sinclair School of Nursing, University of Missouri, Columbia, MO, United States; ^8^Health Management and Informatics, School of Medicine, University of Missouri, Columbia, MO, United States; ^9^Surgery, School of Medicine, University of Missouri, Columbia, MO, United States; ^10^Mathematics, College of Arts and Science, University of Missouri, Columbia, MO, United States

**Keywords:** cardiac function, non-invasive monitoring, mathematical modeling, ballistocardiography, myocardial infarction, ventricular contractility

## Abstract

Left ventricular (LV) catheterization provides LV pressure-volume (P-V) loops and it represents the gold standard for cardiac function monitoring. This technique, however, is invasive and this limits its applicability in clinical and in-home settings. Ballistocardiography (BCG) is a good candidate for non-invasive cardiac monitoring, as it is based on capturing non-invasively the body motion that results from the blood flowing through the cardiovascular system. This work aims at building a mechanistic connection between changes in the BCG signal, changes in the P-V loops and changes in cardiac function. A mechanism-driven model based on cardiovascular physiology has been used as a virtual laboratory to predict how changes in cardiac function will manifest in the BCG waveform. Specifically, model simulations indicate that a decline in LV contractility results in an increase of the relative timing between the ECG and BCG signal and a decrease in BCG amplitude. The predicted changes have subsequently been observed in measurements on three swine serving as pre-clinical models for pre- and post-myocardial infarction conditions. The reproducibility of BCG measurements has been assessed on repeated, consecutive sessions of data acquisitions on three additional swine. Overall, this study provides experimental evidence supporting the utilization of mechanism-driven mathematical modeling as a guide to interpret changes in the BCG signal on the basis of cardiovascular physiology, thereby advancing the BCG technique as an effective method for non-invasive monitoring of cardiac function.

## 1. Introduction

The gold standard for characterizing cardiac function *in vivo* consists of the acquisition of left ventricular (LV) pressure-volume (P-V) loops *via* catheterization under different load conditions. Analysis of the P-V loops provides information about the contractile state of the heart. Major indicators of LV contractility are the end systolic pressure-volume relationship (ESPVR) and the pre-load recruitable stroke work (PRSW) (1). A decrease in the slope of the ESPVR has been demonstrated in human failing hearts with deteriorating cardiac function (i.e., ejection fraction <40%) (2, 3). A flattening of PRSW indicates that increased pre-load produces relatively small increases in stroke work due to a reduced contractility (4). The LV catheterization procedure, however, is very invasive and is accompanied by high risks for the patients, limiting the applicability of synchronous P-V measurements in clinical practice. In this perspective, the development of techniques for estimating major P-V loop features in a non-invasive way could potentially enable a better and more effective way to monitor cardiac function in clinical and in-home settings (5–7).

Ballistocardiography provides a very interesting option for non-invasive cardiac monitoring. Its signal, the ballistocardiogram (BCG), is generated by the repetitive motion of the center of mass of the human body as the blood moves within the circulatory system at each heartbeat (8). Thus, BCG-based sensors monitor the cardiovascular activity by capturing the movement of the whole body, or the associated force, that the blood flow generates. In the recent years, numerous sensors have been proposed to measure the BCG signal non-invasively, including weighing scales, bed sensors, and chair sensors, some of which do not even require direct body contact (9–18). The BCG captures the mechanical and fluid-dynamical properties of the cardiovascular system as a whole, including heart, heart valves, arteries, capillaries, and veins. While most of the BCG-based methods aim at estimating heart rate and respiration rate (19–23), some studies have documented changes in the BCG waveforms associated with specific disease conditions (8, 24) or changes in blood pressure (25–27). To date, however, a clear understanding of how specific changes in cardiovascular function translate into specific changes of the BCG waveform is still lacking, thereby limiting the widespread use of BCG-based techniques for cardiac monitoring.

The main motivation of our investigation is to establish a connection between changes that can be measured non-invasively *via* BCG-based monitoring and changes in cardiac function that are embodied in specific features of the P-V loops measured *via* LV catheterization, such as changes in ESPVR and PRSW. Our strategy is to utilize the mathematical model for cardiovascular function and BCG physiology proposed in (28, 29) to generate hypotheses on how the BCG waveform is expected to change upon changes in LV contractility and then test such hypotheses on a pre-clinical swine model of myocardial infarction (MI) both pre- and post-intervention. By analyzing the properties of P-V loops acquired experimentally and P-V loops simulated *via* the mathematical model, we have identified two major features of the BCG waveform that are predicted to change with LV contractility. These features are *(i)* the relative Timing between ECG and BCG signals, henceforth denoted as TEB, and *(ii)* the amplitude of the BCG signal. The reproducibility of these measurements is also assessed by repeating the data acquisition multiple times on the same swine. Overall, our goal is to develop methods of analysis that enable the interpretation of changes in the BCG signal on the basis of mechanisms of cardiovascular physiology, as we strongly believe that a mechanistic interpretation of changes observed in the signals will be the key to bridge the advances in physiology research with the needs of clinical practice. This work represents a first step in this direction.

## 2. Methods

The starting point of this work is the mechanism-driven model proposed in (28, 29), where the fundamental principles of cardiovascular physiology are translated into mathematical equations by means of the electric analogy to fluid flow. A summary of the model features that are relevant for the present work is provided in Section 2.1. In particular, the model describes the LV pumping action by means of a time-varying elastance. For the isolated heart, i.e., not considered in connection with the vascular system, the maximum of the LV time-varying elastance in the model is determined by a parameter denoted by ${\tilde{E}}_{L}$. Even though it is intuitive that ${\tilde{E}}_{L}$ and LV contractility are related, it remains to be determined how changes in ${\tilde{E}}_{L}$, which characterizes the ventricle in isolation, relate to changes in the LV P-V loops under various loading conditions, which result from the ventricular function in connection with the rest of the vasculature. In order to clarify this relationship, we use the closed-loop cardiovascular model to simulate multiple LV P-V loops obtained under conditions of reducing pre-load through transient occlusion of the inferior vena cava. The transients in LV pressure and volume simulated with the model are qualitatively compared with those acquired *in vivo* on swine, Section 2.2. Next, the model is used as a virtual laboratory to identify specific features of the BCG waveform that are predicted to change as LV contractility is reduced. These features result to be the TEB and the BCG amplitude. Synchronous ECG and BCG data were acquired in a pre-clinical swine model of myocardial infarction (MI) both pre- and post-intervention (ischemia-reperfusion; see Section 2.3) in order to assess the validity of the model predictions. Data acquisitions were also repeated multiple times on the same animal to verify that the changes in TEB and BCG amplitude associated with a major cardiac insult, such as that induced by MI, are substantially larger than the changes associated with natural small fluctuations in cardiac activity. The details of the synchronous ECG and BCG signal acquisition are provided in Section 2.4.

### 2.1. Mechanism-Driven Modeling of Cardiovascular Physiology and BCG-Based Monitoring

**Figure 1:**
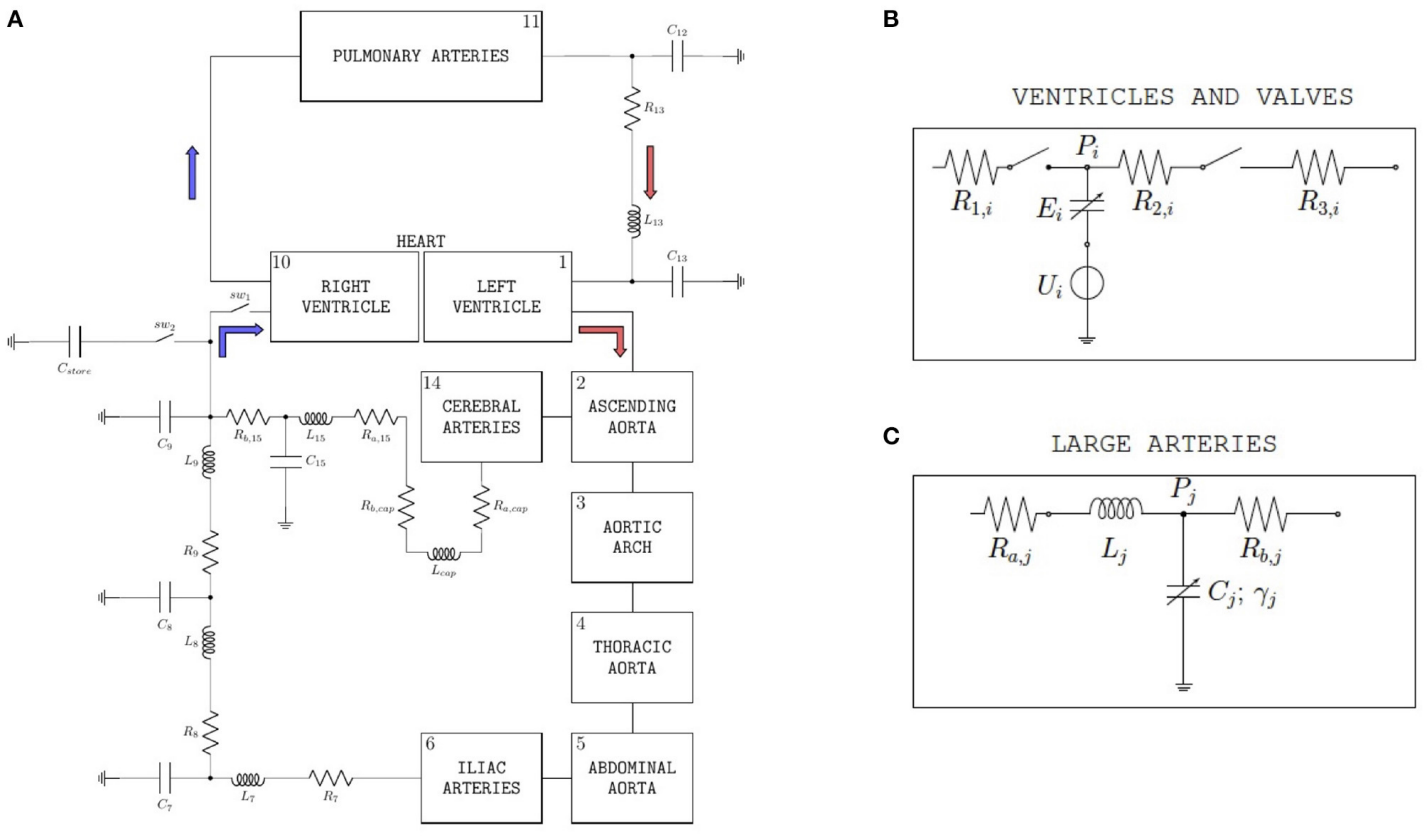
**(A)** Schematic representation of the closed-loop model utilized to simulate blood flow through the cardiovascular system. With respect to the version of the model described in (29), two ideal switches (*sw*_1_ and *sw*_2_) and a capacitor (*C*_*store*_) have been added upstream of the right ventricle in order to simulate the experimental procedure performed on swine to acquire data of left-ventricular pressure and volume. **(B)** Basic model components for ventricles and valves. The subscript *i* is used to distinguish between left (*i* = *L*) and right (*i* = *R*) ventricles; *P*_*i*_ is the ventricular pressure; *R*_1,*i*_, *R*_2,*i*_, and *R*_3,*i*_ are hydraulic resistances assumed to be constant; *U*_*i*_ and *E*_*i*_ characterize ventricular pumping, *E*_*i*_ representing the variable ventricular elastance. *(Bottom)* Large arteries; the subscript *j* is used to distinguish among different arterial segments, specifically aortic arch (*j* = 3), thoracic aorta (*j* = 4), abdominal aorta (*j* = 5), and iliac arteries (*j* = 6); *P*_*j*_ is the pressure of the specific arterial segment; *R*_*a,j*_ and *R*_*b,j*_ are the hydraulic resistances; *L*_*j*_ is the inductance; *C*_*j*_ and γ_*j*_ represent elastic and viscoleastic properties of the arterial wall, respectively. **(C)** Basic model components for large arteries. The subscript *j* is used to distinguish among different arterial segments, specifically aortic arch (*j* = 3), thoracic aorta (*j* = 4), abdominal aorta (*j* = 5), and iliac arteries (*j* = 6); *P*_*j*_ is the pressure of the specific arterial segment; *R*_*a,j*_ and *R*_*b,j*_ are the hydraulic resistances; *L*_*j*_ is the inductance; *C*_*j*_ and γ_*j*_ represent elastic and viscoleastic properties of the arterial wall, respectively.

The model proposed in (29) and schematized in Figure 1A leverages the analogy between electric circuits and hydraulic networks, where electric potentials, electric charges, and electric currents correspond to fluid pressures, fluid volumes, and volumetric flow rates, respectively (29, 30). Since the model translates into mathematical equations the fundamental mechanisms of cardiovascular physiology driving the flow of blood through the cardiovascular system, we refer to this model as *mechanism-driven model* of cardiovascular physiology, henceforth shortened as *cardiovascular model*. We note that, among the numerous mechanism-driven models that have been utilized for blood flow simulations [see e.g., (31)], the one proposed in (29) proved capable of obtaining the BCG waveform starting from the basic principles of cardiovascular physiology and, for this reason, will be used in the present work. The cardiovascular system is represented as a closed-loop, see Figure 1, where the pumping action of the ventricles drives the flow of blood through the systemic and pulmonary circulations. The complete set of equations and parameter values of the cardiovascular model have been reported in (29). In this section, we recap the model features that are particularly relevant for the study at hand.

#### 2.1.1. Model Description of the Ventricles

Each ventricle is modeled *via* a variable capacitor and a voltage source connected in series, see Figure 1B. Normalized activation functions *a*_*L*_(*t*) and *a*_*R*_(*t*) are introduced to describe the mechanical effect of the excitation-contraction coupling for the left and right ventricles, respectively. Following (29), we define the normalized activation functions as follows:


(1) $$\begin{array}{l} a_{i}(t_{m})=\\ \{\begin{array}{cc} \frac{\tanh(5q_{i}\;t_{m})}{\alpha }\;\frac{\tanh(q_{i}(t_{m}-T_{a}))-\tanh(q_{i}(t_{m}-T_{b}))}{2} & \text{for }t_{m}<T_{s}\\ 0 & \text{otherwise} \end{array} \end{array}$$

with *i* = *L, R*, where *t*_*m*_ = mod(*t, T*_*c*_), *T*_*c*_ being the length of the entire cardiac cycle, and *T*_*a*_, *T*_*b*_, and *T*_*s*_ are positive constants representing portions of the cardiac cycle. In Equation (1), α is a normalization constant that allows for the maximum of the activation functions to be equal to 1. For the simulations at baseline conditions reported in this article, we considered *T*_*c*_ = 0.8 s, *T*_*s*_ = 0.34 s, *T*_*a*_ = 0.08 s, *T*_*b*_ = 0.45 s, α = 0.8218, and *q*_*i*_ = 2π s^−1^, for *i* = *L, R*.

The voltage sources in the ventricle schematic of Figure 1B are characterized by the following constitutive equations


(2) $$\begin{array}{r} U_{L}\left(t\right)=ULOa_{L}\left(t\right)\;\text{and }U_{R}\left(t\right)=UROa_{R}\left(t\right) \end{array}$$

where *ULO* and *URO* are positive constants representing the reference peak isovolumic pressures. For the simulations at baseline conditions reported in this article, we considered *ULO* = 50 mmHg and *URO* = 24 mmHg. The variable capacitors in the ventricle schematic of Figure 1B represent time-varying elastances, see, e.g., (32), characterized by the following constitutive equations:


(3) $$\begin{array}{r} E_{L}\left(t\right)=ELD+ELSa_{L}\left(t\right)\;\text{and }E_{R}\left(t\right)=ERD+ERSa_{R}\left(t\right). \end{array}$$

During diastole, *a*_*L*_(*t*) and *a*_*R*_(*t*) are equal to 0 and each ventricle behaves as a chamber of constant elastance denoted by *ELD* and *ERD* for the left and right ventricle, respectively. During systole, the elastance changes with time as modulated by the normalized activation function. For the simulations at baseline conditions reported in this article, we considered *ELS* = 1.375 mmHg ml^−1^, *ELD* = 0.04 mmHg ml^−1^, *ERS* = 0.23 mmHg ml^−1^, and *ERD* = 0.01 mmHg ml^−1^.

#### 2.1.2. LV Elastance, Pressure, Volume, and PRSW

Let us focus on the left ventricle, as it bears particular relevance for the study developed in this work. If the LV were isolated, then the maximum value attained by its elastance would be given by


(4) $$\begin{array}{r} {\tilde{E}}_{L}=ELD+ELS \end{array}$$

and it would occur when the normalized activation function *a*_*L*_(*t*) is equal to 1. We note that ${\tilde{E}}_{L}$ is positive constant that characterizes the intrinsic properties of the ventricular chamber irrespective of its loading conditions. When the ventricle is considered in connection with the rest of the cardiovascular system in the closed-loop model, the LV pressure *P*_*L*_(*t*) and LV volume *V*_*L*_(*t*) can be computed at every time *t*, and their ratio *P*_*L*_(*t*)/*V*_*L*_(*t*) gives the actual instantaneous elastance at time *t*. Thus, the value of ${\tilde{E}}_{L}$ and the maximum value of *P*_*L*_(*t*)/*V*_*L*_(*t*) do not necessarily coincide, since the latter is influenced not only by the intrinsic properties of the ventricular chamber but also by the loading conditions that the ventricle is experiencing at each heart beat [(1)]. The time during the cardiac cycle at which the maximum of *P*_*L*_(*t*)/*V*_*L*_(*t*) occurs marks the end of the systole, as defined by (33), and it will be used to determine the ESPVR in the simulated LV P-V loops reported in Section 3.1. The slope of ESPVR is commonly referred to as end-systolic elastance (Ees).

From the simulated LV P-V loops, the PRSW can be calculated as the slope of the line approximating the relationship between stroke work (SW) and end-diastolic volume (EDV). For each cardiac cycle, SW is calculated as the area under a complete LV P-V loop by means of the following integral


(5) $$\begin{array}{r} \text{SW}=\oint P_{L}dV_{L}. \end{array}$$

whereas EDV is calculated as the maximum of the LV volume *V*_*L*_(*t*) over the considered cardiac cycle.

#### 2.1.3. Model Description of Heart Valves and Vasculature

The heart valves are modeled as ideal switches, see Figure 1B, which close as soon as a positive pressure difference is established between the upstream and downstream nodes of the switch. The large arteries, namely aortic arch, thoracic aorta, abdominal aorta, iliac arteries, and cerebral arteries, share the same mathematical description, see Figure 1C, which includes two resistors, one inductor and one variable capacitor representing hydraulic resistance, inertial effects and wall compliance, respectively. We note that variable capacitors are adopted to capture the viscoelastic properties of the arterial walls, which significantly affect the transmission of the pressure waveform along the arterial tree (34, 35). While linear capacitors are characterized by a simple proportionality between pressure and volume, the variable capacitors utilized in (29) account for a time delay in the pressure-volume relationship characterizing arterial blood flow, which yields the hysteresis loop reported by many *in vivo* studies (36–38).

#### 2.1.4. Model Description of Inferior Vena Cava Occlusion

With respect to the version of the model described in (29), two ideal switches (*sw*_1_ and *sw*_2_) and a capacitor (*C*_*store*_) have been added upstream of the right ventricle, as shown in Figure 1A. The introduction of these new elements is motivated by the need of simulating the procedure of inferior vena cava occlusion performed on swine to acquire *in vivo* data of left-ventricular pressure and volume under varying pre-load conditions, as detailed in Section 2.2. When the flow through the inferior vena cava is unobstructed, the switch *sw*_1_ is closed and the switch *sw*_2_ is open, thereby allowing the blood to flow into the right ventricle. Conversely, when the vena cava is occluded and the flow to the right ventricle is interrupted, the switch *sw*_1_ is open and the switch *sw*_2_ is closed. The blood temporarily accumulates into the capacitor *C*_*store*_, simulating the temporary storage within the tissues, till the occlusion of the inferior vena cava is terminated and normal blood flow is restored. A value of *C*_*store*_ = 10 ml/mmHg has been used for the simulations reported in this study.

#### 2.1.5. Model Simulation of the BCG Waveform

As the volume of blood dynamically redistributes through the various parts of the body at each heart beat, the mass redistributes inside the body as well, thereby leading to a motion of the overall barycenter (or center of mass) of the human body, also known as BCG. In (29), the authors showed how the cardiovascular closed-loop model summarized above can be used to reconstruct the BCG waveform starting from the model-simulated volumes. Let us enote by *V*_*k*_(*t*) the blood volume occupying the cardiovascular compartment *k* at time *t*. Consistent with previous literature (39), (29), the work in (29) showed that the main systolic peaks of the BCG waveform could be obtained by considering the time-varying volumes in 9 cardiovascular compartments, namely LV, RV, ascending aorta, aortic arch, thoracic aorta, abdominal aorta, iliac arteries, pulmonary arteries, and cerebral arteries. Following (8, 29), the force generated by the BCG can be computed as


(6) $$\begin{array}{rc} f_{A}\left(t\right)=\rho_{b}\overset{N}{\underset{k=1}{\sum }}\frac{d^{2}V_{k}}{dt^{2}}\left(t\right)y_{k} & \text{[dyne]} \end{array}$$

where ρ_*b*_ is the blood density, and *y*_*k*_ is the distance between the compartment *k* and the plane of the heart valves and *N* = 9 is the total number of cardiovascular compartments considered in the BCG model. Even though compartments more peripheral to the circulation do not explicitly appear in the BCG calculation of Equation (6), they contribute to determine the *V*_*k*_(*t*) volumes *via* the closed-loop architecture. For example, the vena cava is not included as one of the 9 compartments selected for the BCG calculation, and yet its occlusion leads to a change in the distribution of blood volume across the whole cardiovascular system, including the volumes *V*_*k*_(*t*) explicitly accounted for in Equation (6). We remark that the second derivatives appearing in Equation (6) are applied to the volumes computed with the closed-loop cardiovascular model, which are not affected by noise as the signals acquired experimentally.

#### 2.1.6. Numerical Solution of the Closed-Loop Model

The mathematical model has been implemented in OpenModelica (40) and solved using a differential algebraic system solver, DASSL (41), with a tolerance of 10^−6^ and a time step of 0.001 s, as in (29). A functional mockup unit (FMU) was generated from OpenModelica and utilized in a Python code to expedite simulations for different values of the model parameters. The simulations related to the occlusion of the inferior vena cava (see **Figures 5** and **6**) are performed over a total simulation time interval of 40 s. The first 5 s of simulation time are discarded as they show transients in the solution that are purely due to the numerical algorithm and are not representative of the physiology of the system. The simulated occlusion starts at 20 s and ends at 30 s. The simulations related to the BCG waveform (see **Figure 7**) are performed over a total of 10 cardiac cycles. Only the last 2.5 cycles are reported in the figure. The values of the model parameters that are not explicitly reported here are set to be equal to those already published in (29). We remark that, in this work, the goal is not to adapt the values of the model parameters to the specific subject under consideration, for which a procedure similar to that described in (42) could be applied. Rather, the cardiovascular model is used as a virtual laboratory to theoretically predict which changes in the BCG waveforms are expected to occur as a consequence of reduced LV contractility, and then utilize these predictions to analyze the experimental data in a manner that is informed directly by the physiology of the cardiovascular system.

### 2.2. *In vivo* Study of Cardiovascular Function on Swine

**Figure 2:**
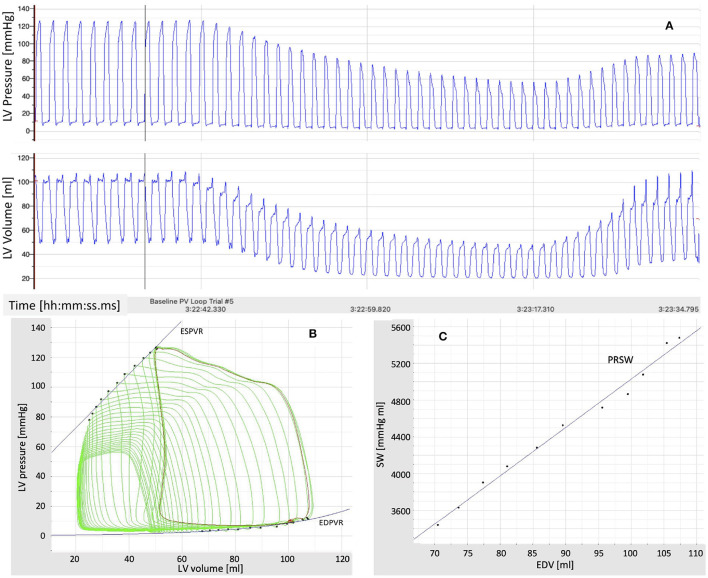
**(A)** Time recordings of pressure and volume in the left ventricle (LV) under conditions of reducing pre-load, achieved through transient occlusion of the inferior vena cava *via* inflation of a balloon catheter. **(B)** Pressure-Volume (P-V) loops in the left ventricle (LV) under conditions of reducing pre-load. The end-systolic and end-diastolic P-V relationships (ESPVR and EDPVR) are also shown. **(C)** Relationship between stroke work (SW) and end-diastolic volume (EDV) under conditions of reducing pre-load. The slope of the line approximating the SW-EDV relationship is the pre-load recruitable stroke work (PRSW).

Hemodynamic data collected from previous studies using *in vivo* pressure-volume (P-V) loop techniques to determine left ventricular function in swine were used to test and validate the cardiovascular model (43–45). The animals were anesthetized with a telazol (5 mg/kg)/xylazine (2.25 mg/kg) mix and maintained on propofol (6–10 mg kg^−1^ min^−1^ with bolus as needed). Heparin was given with an initial loading dose of 300 U/kg IV, followed by maintenance of 100 U/kg each hour. A median sternotomy was performed, and the pericardium was opened near the apex for insertion of the P-V loop catheter. Care was taken to cause minimal disruption to the pericardium. P-V loops were measured utilizing a calibrated 5F admittance-based Advantage catheter (Transonic Systems, Inc.; Ithaca, NY) positioned in the LV *via* a small apical incision. A 14F balloon occlusion catheter (Edward Life Sciences) was advanced to the inferior vena cava at the level of the apex of the heart *via* the deep femoral vein. Catheter placement was visualized and confirmed using angiography (Infimed software). After the insertion of catheters, animals were allowed to stabilize for 10 min until a baseline homeostasis was established. P-V loops were recorded at rest under conditions of reducing pre-load, achieved through transient occlusion of the inferior vena cava *via* inflation of the balloon catheter. A representative recording example from a 1-year old, intact female Ossabaw swine (49 kg) that was previously used as a sedentary control is shown in Figure 2 (44). LV pressure and LV volume recorded as a function of time are reported in Figure 2A. The LV P-V loops and the end-systolic and end-diastolic pressure-volume relationships (ESPVR and EDPVR) are reported in Figure 2B. The relationship between stroke volume (SW) and end-diastolic volume (EDV) is reported in Figure 2C, along with its linear interpolant whose slope is known as pre-load recruitable stroke work (PRSW).

Animals were fed once per day, and water was provided ad libitum. All animal protocols were in accordance with the “Principles for the Utilization and Care of Vertebrate Animals Used in Testing Research and Training” and approved by the University of Missouri Animal Care and Use Committee.

### 2.3. *In vivo* Study of Myocardial Infarction on Swine

**Figure 3:**
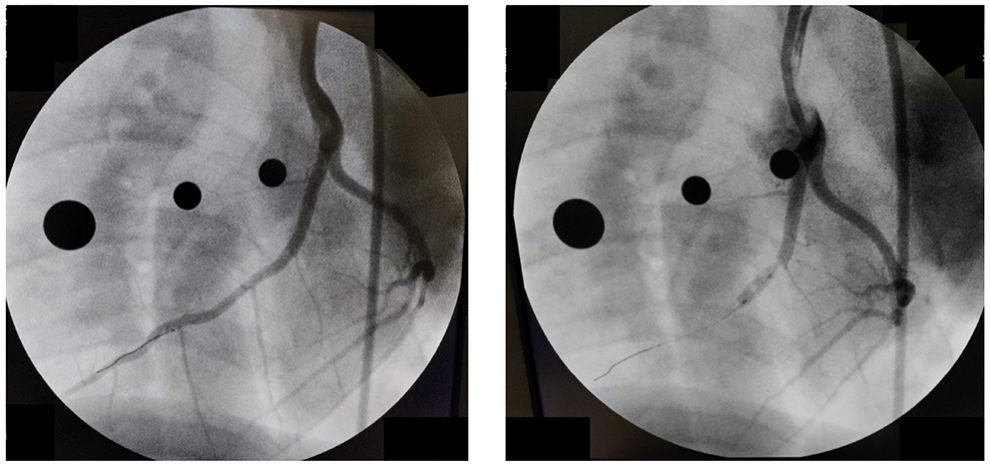
Angiography pre-**(Left)** and post-**(Right)** occlusion of the Left Anterior Descending Coronary artery.

Data acquisition was accomplished in an acute experimental setting of mycardial infarction (MI) pre- and immediately post-ischemia (during reperfusion) in one Yorkshire swine (intact female, 30 kg, approximately 3 months old) in Dr. Emter's lab. The MI was created using an established ischemia-reperfusion protocol with modifications (46). The animal was sedated with 7.0 mg/kg Telazol intramuscular (IM) followed by 0.03 mg/kg buprenorphine IM for pain control. Anesthesia was induced with 2.0 mg/kg propofol intravenous (IV) and maintained by constant rate infusion (CRI) of 4–20 mg/kg. The animal was mechanically ventilated with 100% oxygen at a rate of 8–12 breaths per minute with tidal volume 10–20 mL/kg and 20–25 cm H20 pressure. The left anterior descending (LAD) coronary artery was completely occluded just distal to the 1st diagonal (D1) for 90 min using a balloon angioplasty catheter (3.5 mm x 8 mm, Abbott Trek) followed by reperfusion. Placement of the balloon was accomplished using a 6F guide catheter (Boston Scientific) introduced into the LAD coronary artery under fluoroscopic guidance (see Figure 3). During the ischemia-reperfusion procedure, ECG and vital signs were continuously monitored and the animal was cardioverted to prevent fatal arrhythmia using a standard 200 Joule biphasic defibrillator. Transthoracic M-mode echocardiography was performed pre- and post-MI in the supine/right lateral position as previously described (44, 45, 47–50). Short-axis two-dimensional M-mode images were recorded at the mid-papillary level using a GE Vivid I Ultrasound system with a 2.5-MHz transducer and all analyses were performed offline using GE EchoPac Software. Left ventricular fractional shortening % was calculated from M-mode recordings.

### 2.4. Synchronous Acquisition of ECG and BCG Signals on Swine

**Figure 4:**
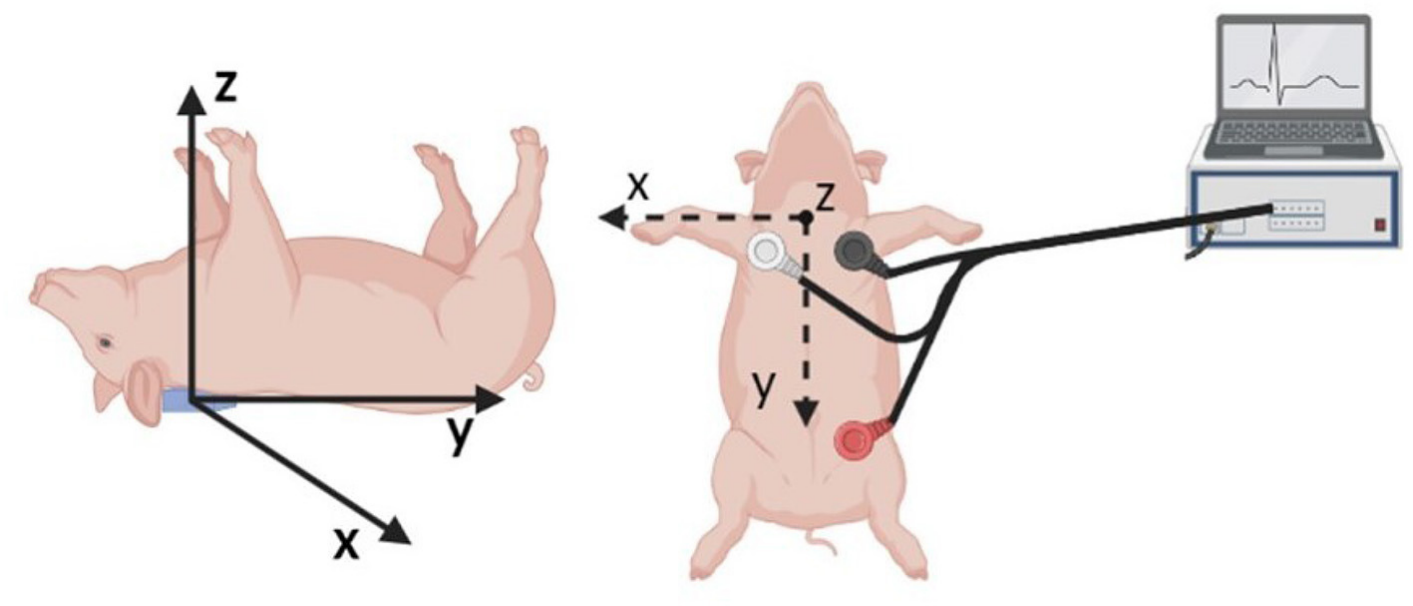
Schematic of the sensor placement for the synchronous acquisition of ECG and BCG signals. A three axis accelerometer positioned under the swine neck with the indicated axis orientation is utilized to acquire the BCG signal **(Left)**. The three leads for ECG acquisition are positioned as illustrated **(Right)**.

An AD Instruments PowerLab Data Acquisition system was utilized for the synchronous acquisition of electrocardiogram (ECG) and BCG. A three lead ECG was acquired at a rate of 200 sample per second, with the leads positioned as shown in Figure 4. The BCG signal was acquired by means of a three-axis accelerometer (Kionix, Inc.) with a sensitivity of 1000 mV/g and a sampling rate of 200 samples per second. The accelerometer was placed under the swine's neck and oriented as shown in Figure 4. The ECG and BCG signals have been filtered *via* a 6th order Butterworth bandpass filter to remove the low frequency respiratory motion and the high frequency noise. Cut-off frequencies of 0.7–40 Hz and 1.25–15 Hz have been used for the ECG and BCG signals, respectively. The choices for the sampling rates and the cut-off frequencies adopted in this work result from the extensive work on ECG and BCG signals conducted within the Center for Eldercare and Rehabilitation Technology at the University of Missouri (18, 25, 51).

Synchronous ECG-BCG acquisitions were performed continuously for 3 min. For the Yorkshire swine who underwent the ischemia-reperfusion procedure for MI induction illustrated in Section 2.3, a total of two 3-min acquisition sessions were performed, one pre-MI and one post-MI. In order to assess the repeatability of the feature extraction from the ECG-BCG acquisitions, three additional swine were considered. For these animals, three consecutive 3-min sessions of data acquisition were performed. A time interval of 2 min was allotted between sessions. Table 1 summarizes the information on the swine involved in this work.

**Table 1:** Summary of the information on the swine involved in this work.

**Subject**	**Type**	**Sex**	**Age [months]**	**Weight [kg]**	**Data acquisitions for this study**
A1	Ossabaw	Female	12	49	P-V loops with transient occlusion of the inferior vena cava
A2	Yorkshire	Female	3	30	Synchronous ECG-BCG and Echocardiogram pre- and post-MI
A3	Yorkshire	Female	3	20	Synchronous ECG-BCG and Echocardiogram pre- and post-MI
A4	Yorkshire	Female	3	22	Synchronous ECG-BCG and Echocardiogram pre- and post-MI
A5	Ossabaw	Female	12	62	Synchronous ECG-BCG
A6	Ossabaw	Female	12	60	Synchronous ECG-BCG
A7	Ossabaw	Female	10	52	Synchronous ECG-BCG

## 3. Results

The model simulations of LV P-V loops under conditions of reducing pre-load are presented in Section 3.1, along with the study of the relationship between ${\tilde{E}}_{L}$ and two indicators of LV contractility, namely the ESPVR slope and PRSW. The experimental BCG waveforms acquired on a swine pre- and post-MI are presented in Section 3.2, along with their comparison with the model predicted BCG changes. Finally, in Section 3.3 we present the results of BCG acquisitions in consecutive sessions on three swine, showing that the BCG changes due to the natural fluctuations in cardiac activity are markedly smaller than those due to a major cardiac insult, such as MI.

### 3.1. PV Loops and Ventricular Contractility

The closed-loop model summarized in Section 2.1 proved capable of generating physiologically-reasonable pressure and volume waveforms through the cardiovascular system, as published in (29). Furthermore, the BCG waveform reconstructed from the simulated volumes by means of Equation (6) was successfully validated, both qualitatively and quantitatively, against data published by other research groups as well data on healthy human subjects acquired within the Center for Eldercare and Rehabilitation Technology at the University of Missouri (29). Model simulations on how the BCG signal is expected to change in the cases of heart failure with reduced or preserved ejection fraction were also reported in (29). The insights from these simulations were also leveraged in a case study on the use of sensor signals for the early detection of heart failure (52). The work in (42) showed how personalized values of the model parameters can be learned from non-invasive ECG and BCG measurements by combining the closed-loop model with an evolutionary algorithm. The physiological relevance of the estimated personalized values was evaluated a posteriori by comparing the model-predicted blood pressure with the blood pressure measured by a cuff placed on the arm of the subjects. Previous published work, however, did not consider simulations under varying loading conditions, such as those associated with occlusion of the inferior vena cava. These simulations are important to evaluate whether the cardiovascular closed-loop model is capable of capturing the overall behavior displayed by the experimental curves acquired *in vivo*, such as those reported in Figure 2. Furthermore, these simulations allow to assess the relationship between the maximum value of the elastance ${\tilde{E}}_{L}$ characterizing the isolated LV model and two major indicators of LV contractility (ESPVR slope and PRSW) that are obtained from the LV P-V loops after coupling the LV with the rest of the cardiovascular system.

**Figure 5:**
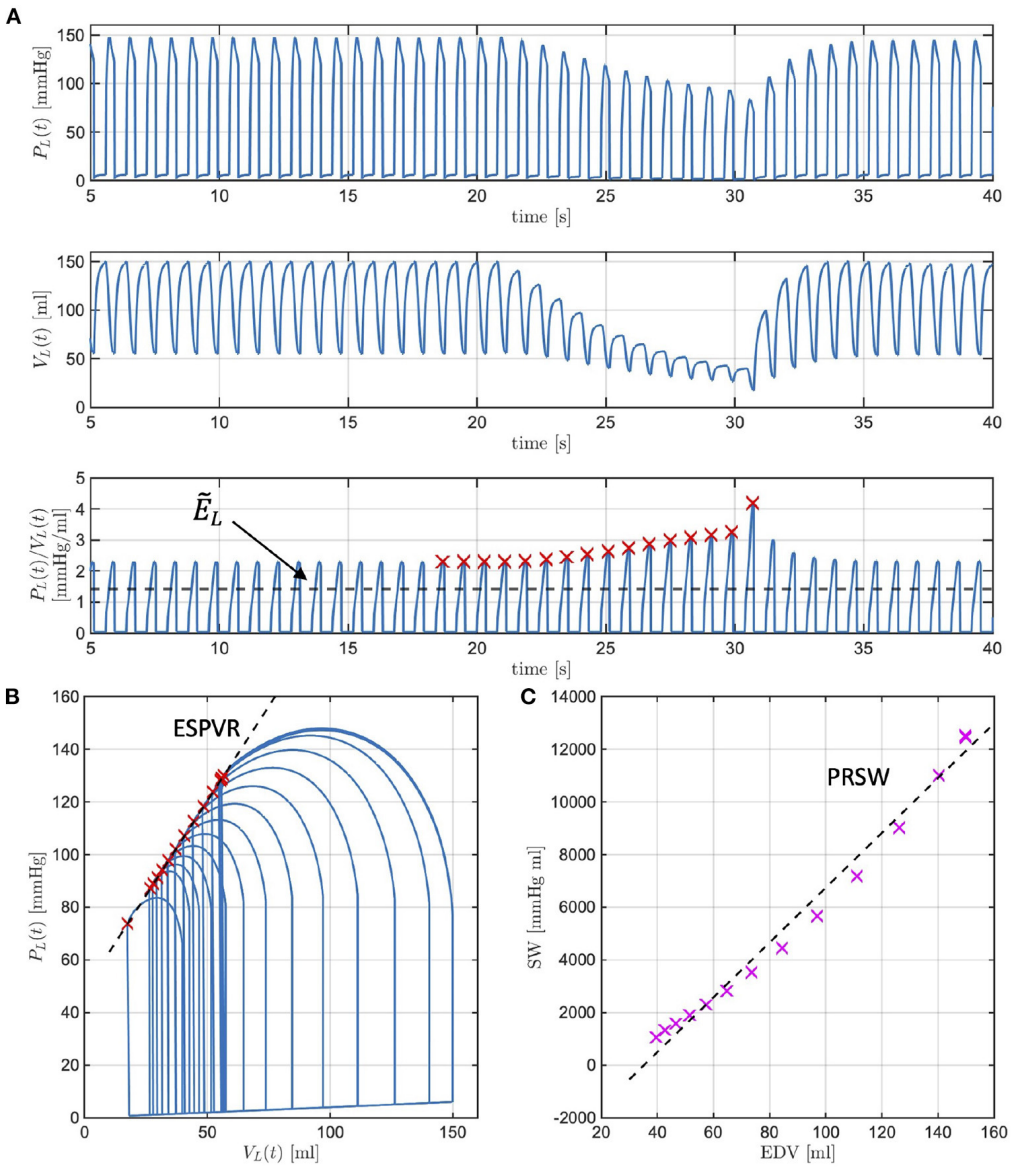
Model-simulated pressure *P*_*L*_(*t*) and volume *V*_*L*_(*t*) in the left ventricle (LV), along with their ratio *P*_*L*_(*t*)/*V*_*L*_(*t*) representing the actual istantaneous elastance **(A)**. The value of ${\tilde{E}}_{L}$ is independent of the loading conditions (black dashed line), whereas the actual instantaneous elastance varies as the load changes. The times at which the actual instantaneous elastance attains its maximum within a cardiac cycle are noted (red crosses) and mark the end-systolic points in the P-V loops whose interpolation gives the end-systolic pressure-volume relationship (ESPVR) **(B)**. The stroke work (SW) computed for each cardiac cycle is reported against the end-diastolic volume (EDV), whose interpolation determines the pre-load recruitable stroke work (PRSW) **(C)**.

Figure 5 reports the model simulations corresponding to the occlusion of the inferior vena cava obtained when all model parameters are considered to be at their baseline values. Figure 5A shows the model-simulated pressure *P*_*L*_(*t*) and volume *V*_*L*_(*t*) in the left ventricle, along with their ratio *P*_*L*_(*t*)/*V*_*L*_(*t*) representing the actual istantaneous elastance. Similarly to what displayed by the experimental curves of Figure 2, the simulated curves show a decrease in the peak pressure accompanied by a decrease in both end-diastolic and end-systolic volumes. The actual instantaneous elastance varies as the load changes, whereas the value of ${\tilde{E}}_{L}$ is independent of the loading conditions and is indicated by an horizontal black dashed line. The times at which the actual instantaneous elastance attains its maximum within a cardiac cycle are noted (red crosses) and mark the end-systolic points in the P-V loops whose interpolation gives the end-systolic pressure-volume relationship (ESPVR), as shown in Figure 5B. For each cardiac cycle, the stroke work (SW) computed *via* Equation (5) is reported against the end-diastolic volume (EDV). The slope of the line interpolating the SW-EDV relationship gives the pre-load recruitable stroke work (PRSW), as shown in Figure 5C.

**Figure 6:**
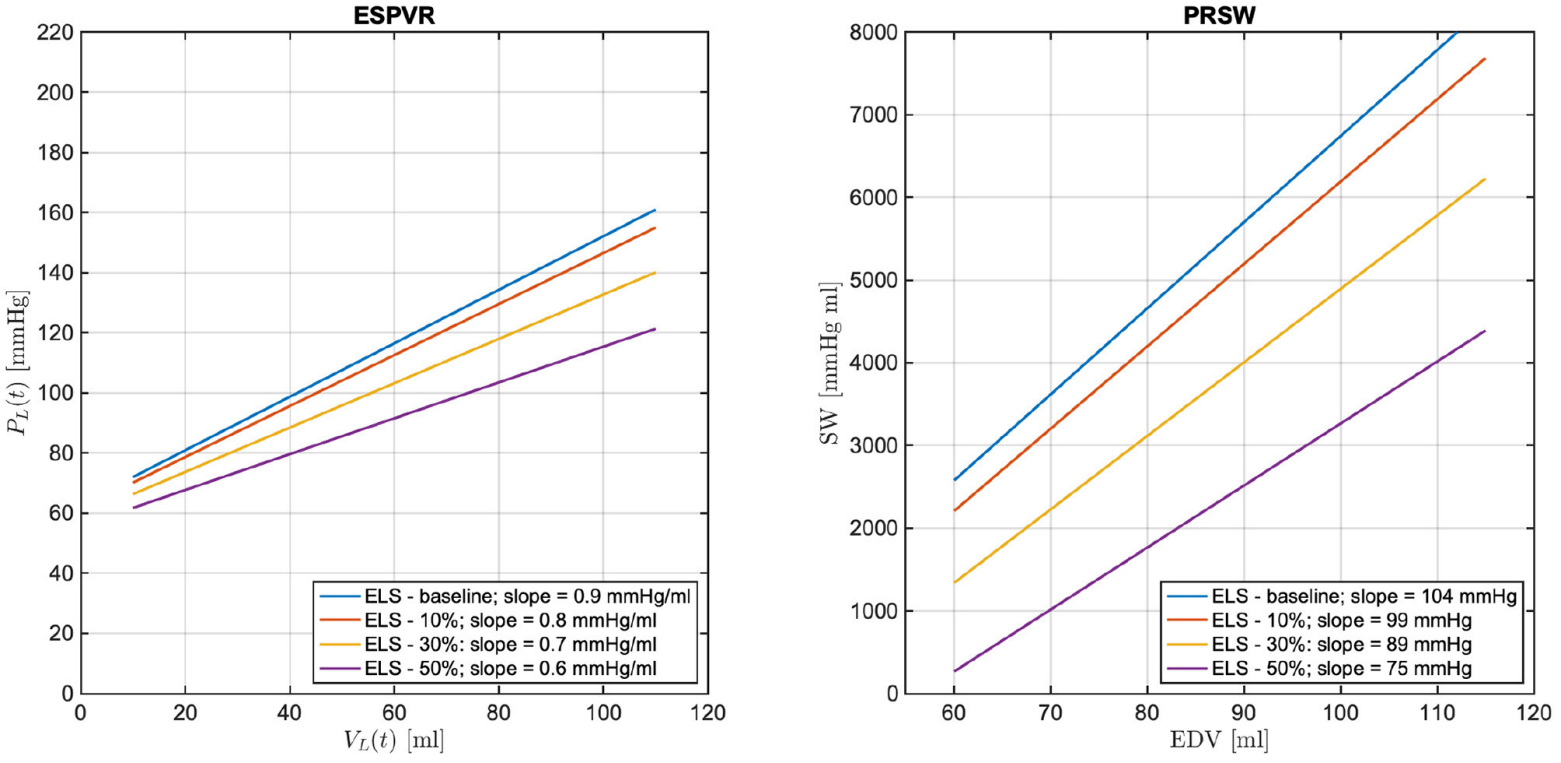
Model simulated end-systolic pressure-volume relationship (ESPVR) **(left)** and pre-load recruitable stroke work (PRSW) **(right)** for *ELS* at baseline (blue line) and for *ELS* reductions of 10, 30, and 50% from baseline (red, yellow, and purple curves, respectively). The numerical values of the ESPVR slopes and PRSW are also reported.

Many studies have evidenced that a decrease in LV contractility manifests in the LV P-V loops, leading to a decrease in the ESPVR slope and a decrease in PRSW. While intuitive that a decrease in ${\tilde{E}}_{L}$ would give rise to similar changes, such conjecture must be verified with simulations. We recall that ${\tilde{E}}_{L}=ELD+ELS$, where *ELD* and *ELS* are positive constants characterizing the ventricle in isolation. By decreasing *ELS* we will be simulating reductions in ${\tilde{E}}_{L}$ due to compromised ventricular properties during systole. The ESPVR lines simulated with the model for *ELS* at baseline (blue line) and for *ELS* reductions of 10, 30, and 50% from baseline (red, yellow, and purple curves, respectively) are reported in Figure 6 (left). As *ELS* is reduced, the slope of the simulated ESPVR lines decreases. We note that when ELS is reduced of the amount specified above, Equation (4) implies that ${\tilde{E}}_{L}$ is equal to 1.4, 1.3, 1.0, and 0.7 mmHg/ml, whereas the ESPVR slope, also referred to as Ees in the physiology literature, obtained from the analysis of the corresponding P-V loops is equal to 0.9, 0.8, 0.7, and 0.6 mmHg/ml. Thus, the maximum value of ventricular elastance ${\tilde{E}}_{L}$ when the ventricle is considered in isolation is higher than the value of Ees actually attained when the ventricle is coupled with the rest of the cardiovascular system. Similarly, the relationships between SW and EDV simulated with the model for *ELS* at baseline (blue line) and for *ELS* reductions of 10, 30, and 50% from baseline (red, yellow, and purple curves, respectively) are reported in Figure 6 (right). As *ELS* is reduced, the slope of the simulated SW-EDV lines, namely the PRSW, also decreases. Specifically, a 50% decrease in *ELS* results in a 33% decrease in ESPVR slope and a 28% decrease in PRSW. These results provide evidence that changes in ${\tilde{E}}_{L}$ due to *ELS* reductions lead to changes in the LV PV-loops that are typical of reduced LV contractility.

**Figure 7:**
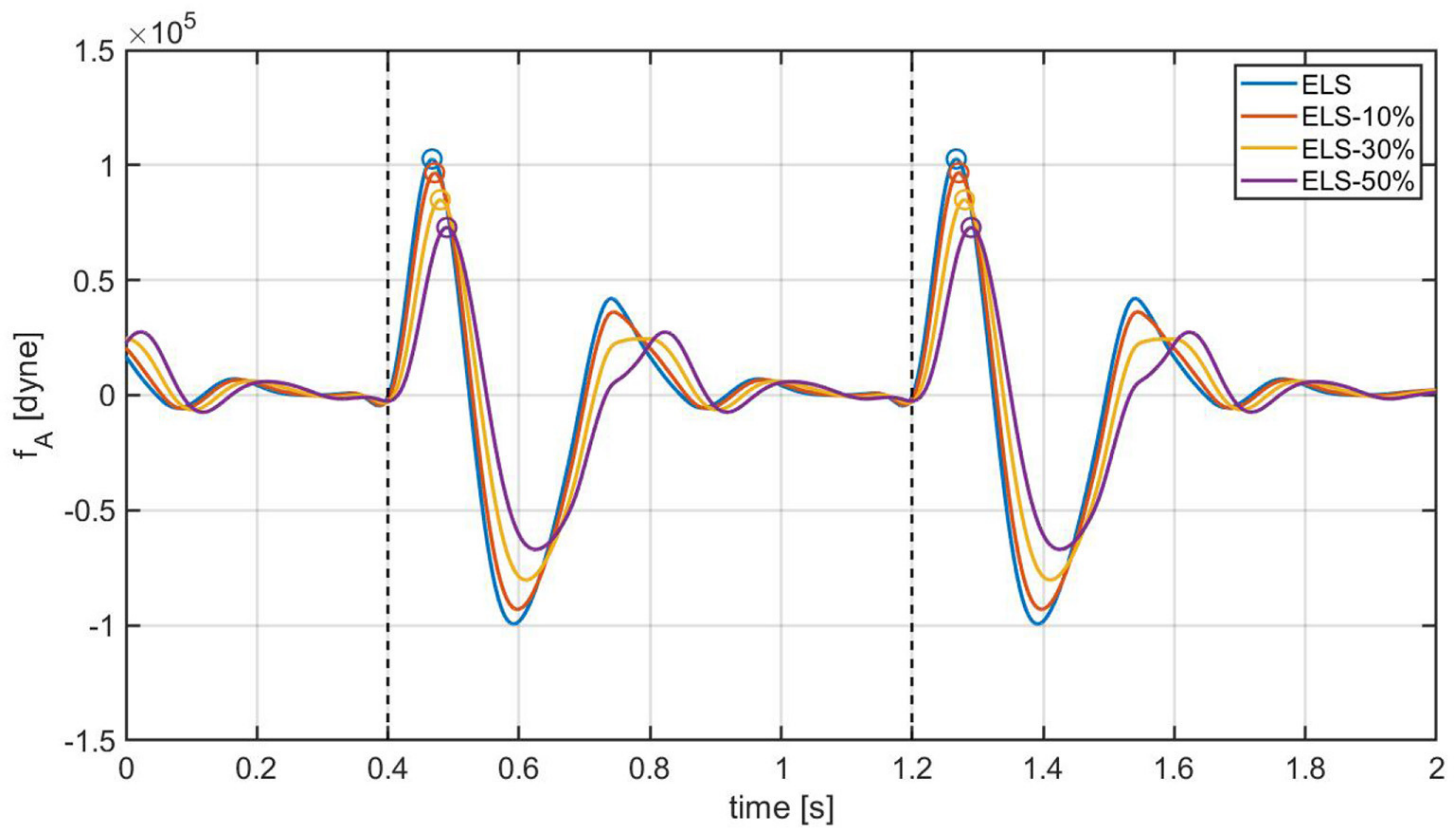
Model-predicted BCG waveforms *f*_*A*_ for different values of the *ELS* parameter. The vertical dashed lines represent the beginning of the systole and the circles indicate the J peaks in the BCG.

As a next step, the cardiovascular model is utilized to predict how changes in ${\tilde{E}}_{L}$ due to *ELS* reductions would manifest in the BCG waveform. Figure 7 shows the model-predicted BCG waveform defined as *f*_*A*_(*t*) in Equation (6) obtained for *ELS* at its baseline value (blue curve) and 10, 30, and 50% reductions from baseline (red, yellow, and purple curves, respectively). Figure 7 displays the model simulations for two consecutive cardiac cycles. The dashed vertical lines mark the beginning of the systole, corresponding to *t*_*m*_ = 0 in the definition of the activation function in Equation (1). A feature of particular relevance in the BCG waveform is the most prominent peak in the systolic part of the cardiac cycle, also known as the J peak (8). The location of the J peaks for decreasing values of *ELS* are marked by circles in Figure 7. The model predicts that a reduction in *ELS* leads to (1) an increase in the time delay between the beginning of the systole (vertical dashed lines) and the J peak (circles); and (2) a decrease in amplitude of the J peak. In order to assess whether these model predictions bear any physiological significance, we examine the changes in the BCG waveform acquired in swine pre- and post-MI, as illustrated in the next section.

### 3.2. BCG Signals Pre- and Post-acute Myocardial Infarction

**Figure 8:**
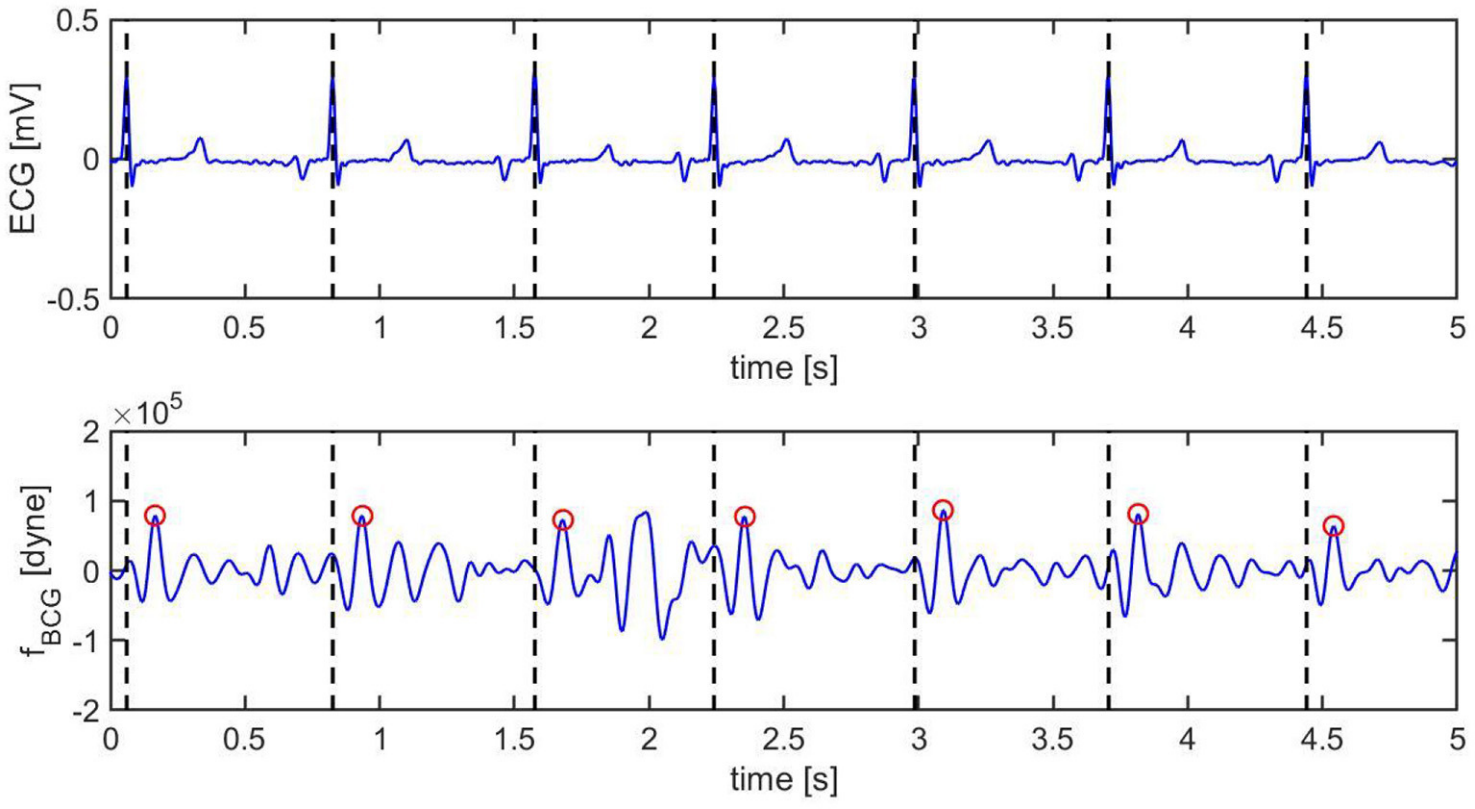
Ten s sample of ECG (top) and BCG (bottom) signals recorded synchronously in swine *before* inducing myocardial infarction (Subject A2). The vertical black dashed lines mark the R peaks in the ECG. The red circles mark the J peak in the BCG.

**Figure 9:**
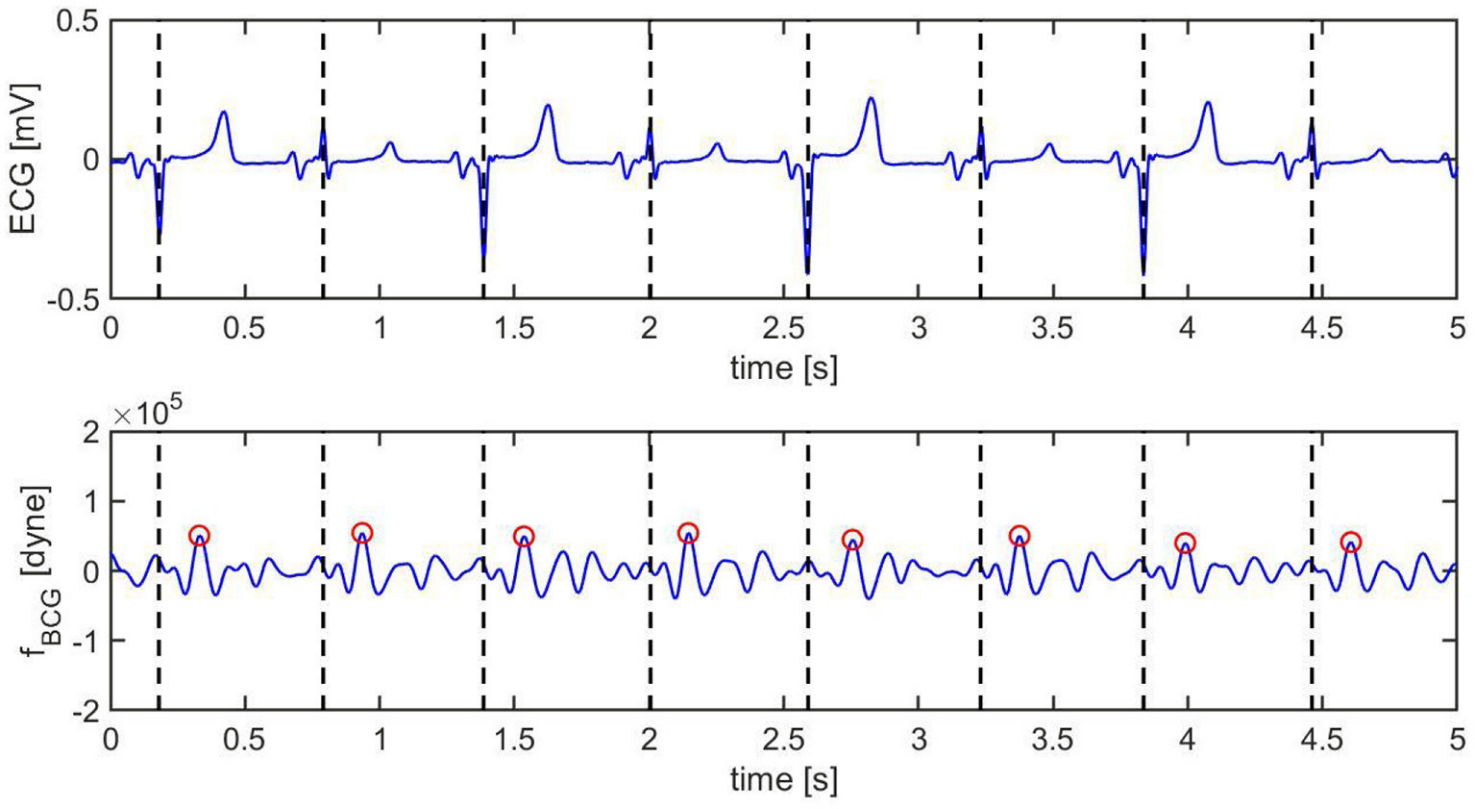
Ten s sample of ECG (top) and BCG (bottom) signals recorded synchronously in swine *after* inducing myocardial infarction (Subject A2). The vertical black dashed lines mark the R peaks in the ECG. The red circles mark the J peak in the BCG.

Figures 8 and 9 show a 10 s sample of ECG and BCG signals recorded synchronously in a swine pre- and post-MI (Subject A2), respectively. It is important to emphasize that, despite being closely related, the BCG waveform measured experimentally, henceforth referred to as *f*_*BCG*_, is not the exact counterpart of the model-predicted BCG waveform denoted as *f*_*A*_. On the one hand, the model-predicted waveform *f*_*A*_ corresponds to the BCG signal acquired on human subjects in the head-to-toe direction, as described and validated in (29). On the other hand, the experimental waveform *f*_*BCG*_ is obtained from the accelerometer placed under the swine neck, where the floor-to-ceiling (and not the head-to-tail) direction provides the stronger signal among the three acquisition axes. We remind that the floor-to-ceiling direction corresponds to the *z*-axis, as shown in Figure 4. Next, a bandpass filter (see Section 2.4) is applied to the floor-to-ceiling acceleration and a multiplicative factor of −1 is introduced in order to account for the action-reaction principle. Finally, the signal is multiplied by the swine mass (30 Kg for the animal under consideration) in order to obtain the experimental BCG waveform *f*_*BCG*_ in the units of dyne.

Pre-MI, the ECG signal appears to be very regular, with strong R peaks marked with vertical black dashed lines in Figure 8 (top). The J peaks in *f*_*BCG*_ are clearly identifiable as the most prominent peaks after the beginning of systole and are marked in red circles in Figure 8 (bottom). Post-MI, the ECG signal shows many irregulaties, such as R peak inversions and a QS morphology in many of the ECG complexes, as shown in Figure 9 (top). The time intervals between consecutive R/QS peaks is visibly reduced with respect to the pre-MI recording. This is indicative of an increase in heart rate, which increased from 82 beats per minute pre-MI to 92 beats per minute post-MI. The J peaks are still present in the BCG, but they are markedly less prominent when compared to the pre-MI case.

**Figure 10:**
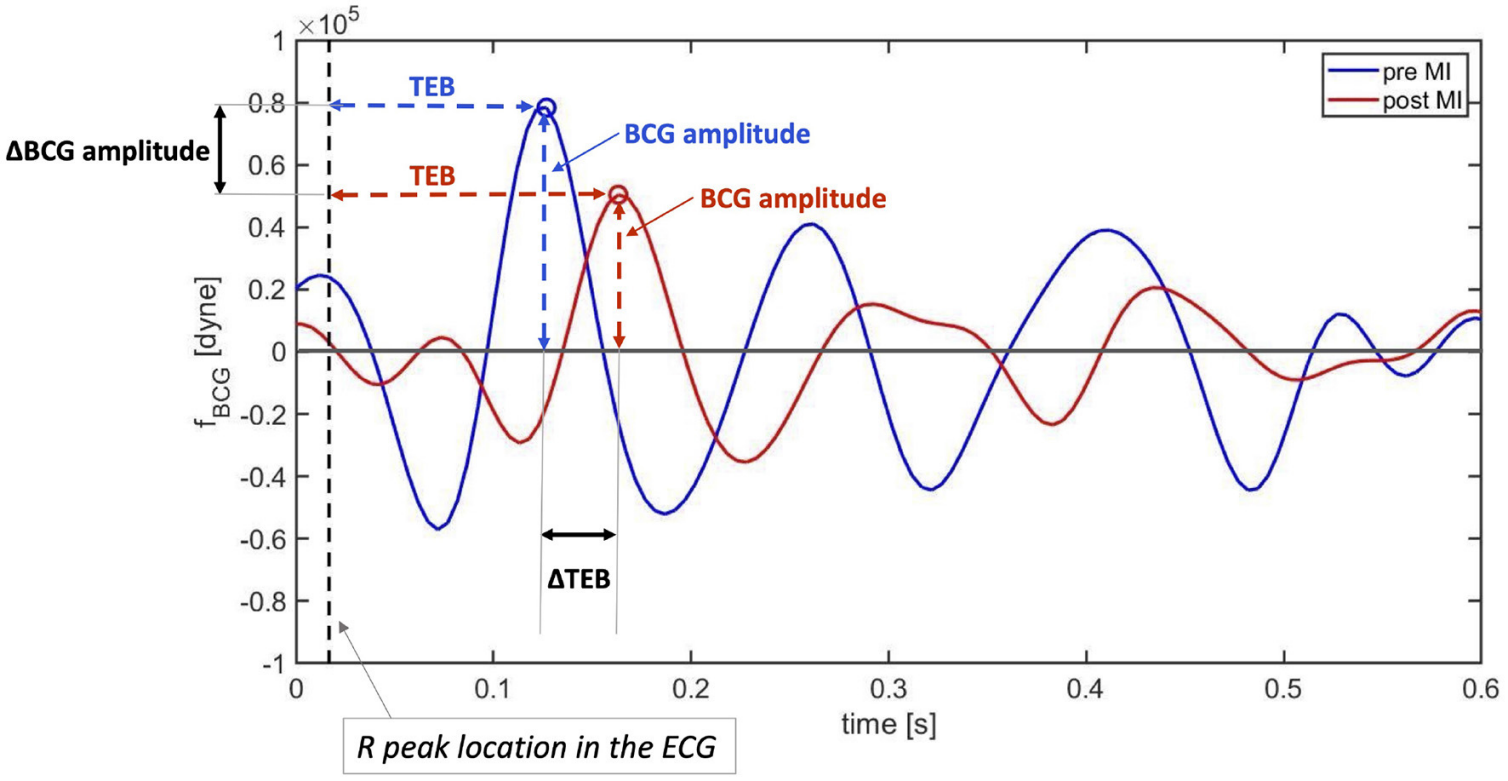
Comparison between BCG waveforms measure before (blue) and after (red) myocardial infarction on a swine. The curves have been aligned with respect to the preceding R peaks in the ECG (vertical dashed line).

A visualization of the changes in the BCG waveform pre- and post-MI is reported in Figure 10. The time location of the preceding R peak (vertical dashed line) has been utilized to align the two curves with respect to the time axis. The location of the J peaks has been marked with circles. The figure shows that the time interval between the ECG and the BCG signals, which we refer to as TEB, is larger post-MI than pre-MI. This is evidenced by the fact that the red circle is located further away from the vertical dashed line than the blue circle. Furthermore, the amplitude of the BCG signal is smaller post-MI than pre-MI. This is evidenced by the fact that the red circle is lower than the blue circle. While not conclusive, this single wave comparison is very informative and allows for easy comparison between experimental data (Figure 7) and model predictions (Figure 10). The increase in TEB and the reduction in BCG amplitude predicted by the mathematical model in the case of a deterioration of cardiac function are consistent with the experimental findings pre- and post-MI.

**Figure 11:**
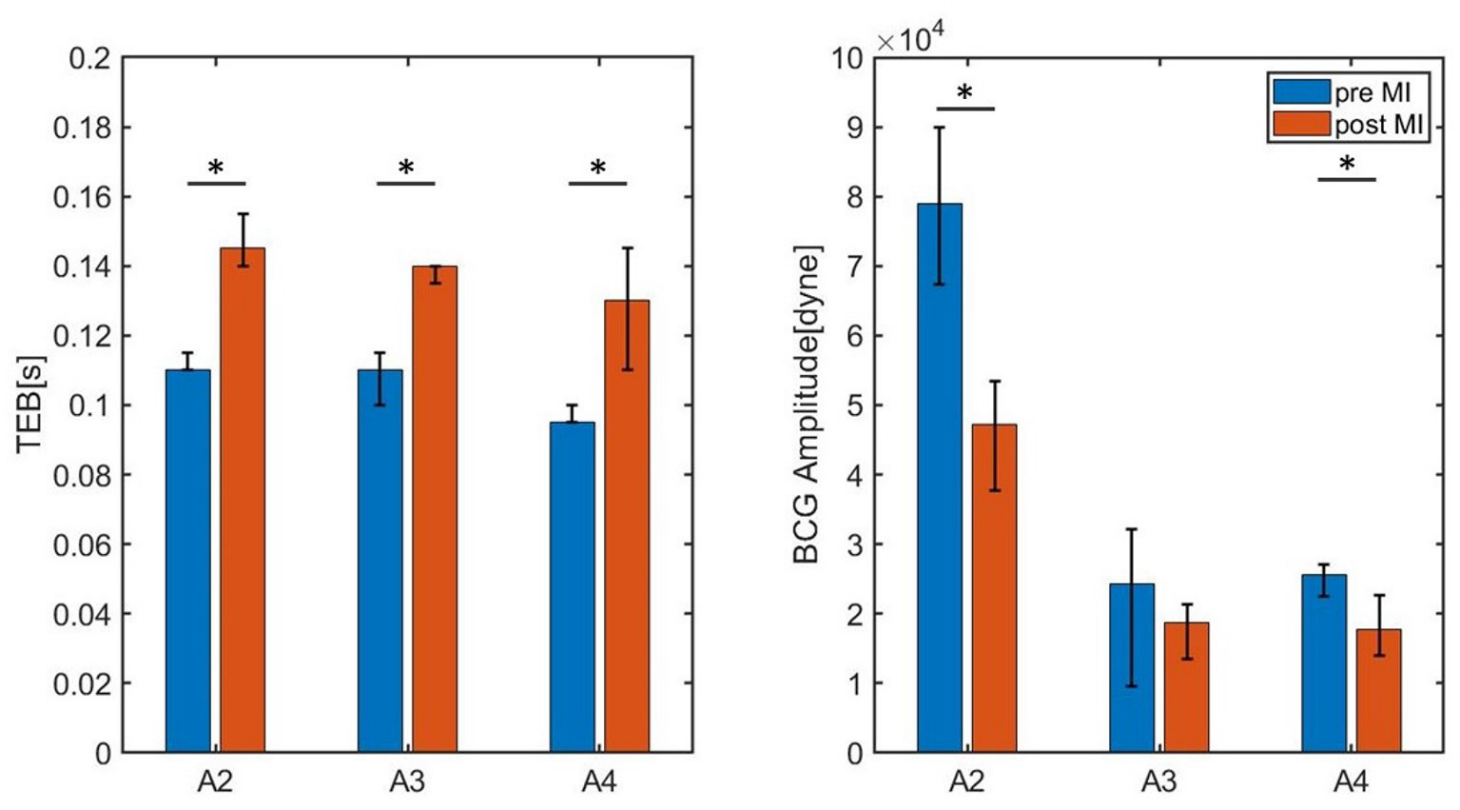
Bar chart summarizing the values of TEB and BCG amplitude for the three swine (Subjects A2, A3, and A4) measured pre- and post-MI. The height of the bars correspond to the medians, whereas the black brackets mark the 25th and 75th percentiles. * P < 0.05 pre-MI vs. post-MI.

These promising findings have been further examined by acquiring data on two additional subjects (Subjects A3 and A4). The values of TEB and BCG amplitude measured for Subjects A2, A3, and A4 pre- and post-MI are reported in Figure 11. The heights of the bars correspond to the medians, while the black brackets indicate the 25th and 75th percentiles. All three subjects exhibit an increase in TEB and a decrease in BCG. Interestingly, the difference in TEB pre- and post-MI is quite consistent among subjects also from the quantitative view point, amounting to 0.035 s, 0.030 s, and 0.035 s, for subjects A2, A3, and A4, respectively. While exhibiting a consistent decreasing trend for all subjects, the BCG amplitude pre- and post-MI does not decrease of the same amount among subjects, with differences of 3.638·10^4^ dyne, 0.5580·10^4^ dyne, and 0.7920·10^4^ dyne for subjects A2, A3, and A4, respectively. The statistical significance of these differences was analyzed with a Mood's Median Test. The differences in TEB were found to be statistically significant with p values less than 0.05. Conversely, the differences in BCG amplitude were found to be statistically significant (*p* < 0.05) only for Subject A2 and A4.

Table 2 reports the median values of TEB and BCG amplitude pre- and post-MI, along with the fractional shortening percent determined by echocardiography and the heart rate determined *via* electrocardiography. All subjects experienced a similar percent decrease in fractional shortening, amounting to 47, 45, and 44 in subjects A2, A3, and A4, respectively. The change in heart rate pre- and post-MI, on the other hand, differs among subjects. For Subject A3 the heart rate post-MI is only slightly higher than pre-MI, while Subjects A2 and A4 experienced and increase of 20 and 41%, respectively. These differences in heart rate pre- and post-MI, along with differences in weight of the subjects (see Table 1), could be contributing factors to the different amounts by which the BCG amplitude has decreased pre- and post-MI. Data acquisitions on a larger subject cohort is currently under way to better address these questions.

**Table 2:** Summary of the median values of TEB and BCG amplitude pre- and post-MI, along with the fractional shortening percent determined by echocardiography and the heart rate determined *via* electrocardiography for Subjects A2, A3, and A4.

		**A2**		**A3**		**A4**	
	**Units**	**PRE**	**POST**	**PRE**	**POST**	**PRE**	**POST**
TEB	s	0.110	0.145	0.110	0.140	0.095	0.130
BCG Amplitude	10^4^ dyne	8.364	4.726	2.430	1.872	2.560	1.768
Fractional Shortening	%	36	19	44	24	45	25
Heart Rate	beat/min	82	99	93	99	78	110

### 3.3. Feasibility of BCG Feature Extraction for Cardiac Function Monitoring

The results illustrated in the previous sections provided evidence that two specific features of the BCG waveform, namely TEB and BCG amplitude, which were predicted by a closed-loop cardiovascular model to be markers for reductions in LV contractility, actually bear high significance for non-invasive monitoring of cardiac function. In this section, we explore the feasibility of extracting these BCG features in a reliable manner. To this end, three sessions of synchronous ECG-BCG data acquisition, each lasting 3 min, are conducted on three different swine, namely A5, A6, and A7, whose information are listed in Table 1. While some variations in TEB and BCG amplitude may exist among sessions, as the cardiac activity is never really a fixed constant for any individual, we expect the variations within consecutive sessions for the same subject to be markedly smaller than the variations reported pre- and post-MI, which is a major insult to cardiac function.

**Figure 12:**
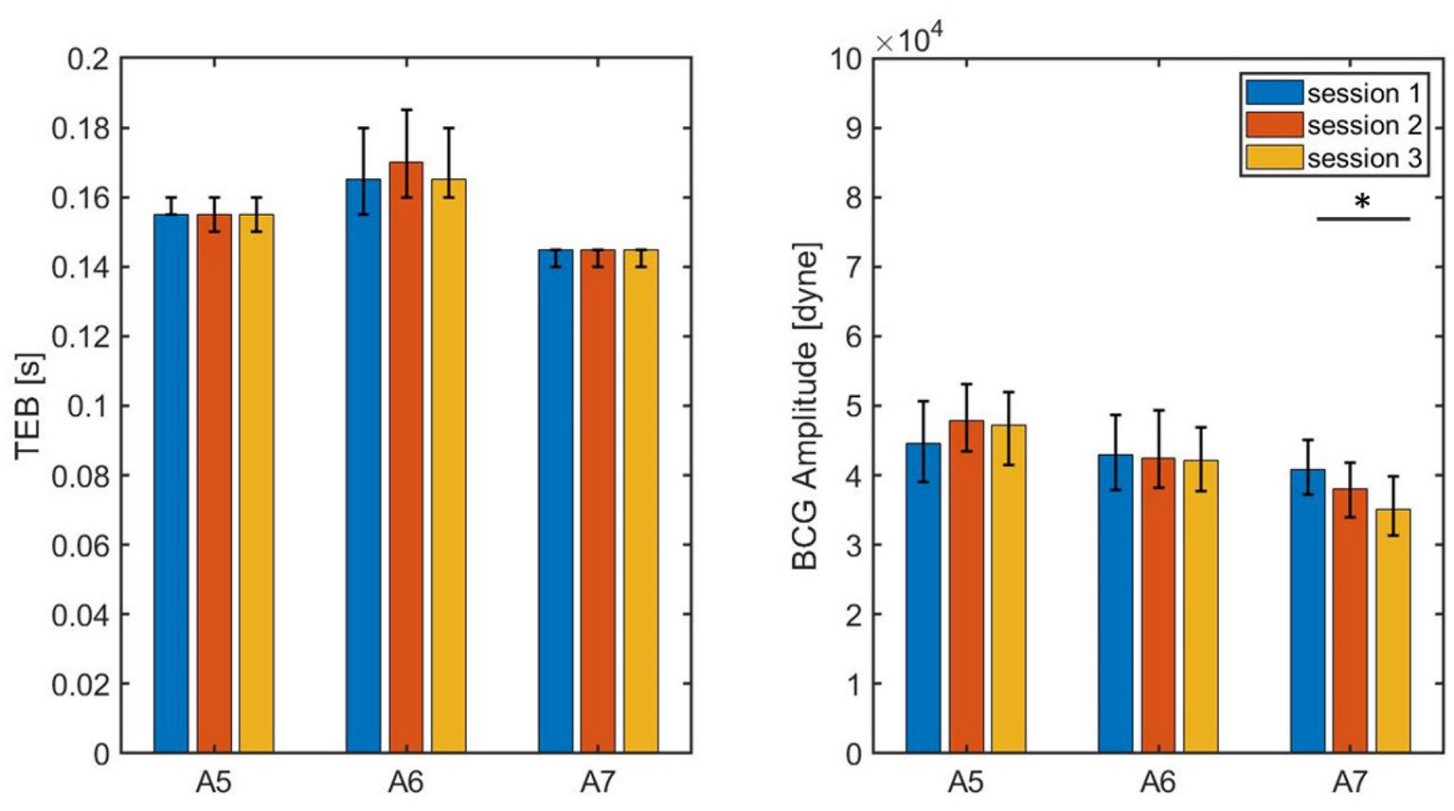
Bar chart summarizing the values of TEB and BCG amplitude measured on three subjects during three consecutive sessions of data acquisition, each lasting 3 min. The bar height correspond to the medians, the black lines correspond to the 25^*th*^ and 75^*th*^ percentiles.* P < 0.05 among sessions.

The values of TEB and BCG amplitude obtained for each session of each swine are summarized in Table 3, with the median values reported along with the 25th and 75th percentiles. The results are also visualized in Figure 12, where the height of the bars correspond to the median values and the black brackets indicate the 25th and 75th percentiles. Overall, the median values of TEB and BCG amplitude appear to be consistent over the three sessions for a given subject. The small differences reported within sessions of the same subject were found not to be statistically significant under the Mood Median Test. The largest variations in TEB are observed in subject A6, where the median is 0.165 s in session 1 and 0.170 s in session 2, with an overall change of 0.005 s. The smallest change in TEB pre- and post-MI was 0.03 s for Subject A3, thus 6 times larger than the largest variation across sessions. Let us now consider the BCG amplitude, whose largest variations are observed in Subject A7. For this subject, the median is 4.085·10^4^ dyne in session 1 and 3.506·10^4^ dyne in session 3, with an overall change of 0.579·10^4^ dyne. The smallest change in BCG amplitude pre- and post-MI was 0.5580·10^4^ dyne, which is comparable to the amplitude variation range reported across sessions. Thus, even though the BCG amplitude is reduced after MI as predicted by the closed-loop cardiovascular model, it may be less reliable as a stand-alone marker for LV contractility since its physiological fluctuations within the same subject are comparable in magnitude to the changes due to a major cardiac insult.

**Table 3:** Median, 25th and 75th percentiles for TEB, representing the time delay between ECG and BCG waveforms, and the BCG amplitude measured on each subject in three consecutive acquisition sessions, each lasting 3 minutes.

	**TEB [**s**]**			**BCG amplitude [** **10^**4**^***dyne***]**		
**Subject**	**Session 1**	**Session 2**	**Session 3**	**Session 1**	**Session 2**	**Session 3**
A5	0.155 (*0.155-0.160*)	0.155 (*0.150-0.160*)	0.155 (*0.150-0.160*)	4.462 (*3.904-5.063*)	4.787 (*4.340-5.319*)	4.727 (*4.150-5.194*)
A6	0.165 (*0.155-0.180*)	0.170 (*0.160-0.185*)	0.165 (*0.160-0.180*)	4.297 (*3.793-4.869*)	4.243 (*3.826-4.929*)	4.222 (*3.773-4.683*)
A7	0.145 (*0.140-0.145*)	0.145 (*0.140-0.145*)	0.145 (*0.140-0.145*)	4.085 (*3.725-4.510*)	3.801 (*3.389-4.187*)	3.506 (*3.137-3.978*)

## 4. Conclusions and Perspectives

This work aimed at establishing a connection between changes in BCG signals, changes in the P-V loops and changes in cardiac function. Theoretical predictions based on a mechanism-driven cardiovascular model have been compared with *(i)* experimental P-V loops in swine, with the goal of assessing the capability of model to simulate LV P-V loops under different loading conditions and establish a relationship with LV contractility, and with (ii) BCG measurements pre- and post-MI, with the goal of assessing the capability of the model to capture changes associated with a deterioration in cardiac function. Overall the results are quite encouraging, as the changes in the BCG waveform observed pre- and post-MI in three swine provide evidence in support of the model predicted BCG changes upon a decrease in LV contractility. Furthermore, the system for BCG measurement utilized in this work is tested during repeated prolonged data acquisitions on three additional swine. Physiological fluctuations in TEB resulted to be 6 times smaller than the changes due to MI, and this yields promise as a specific marker for changes in LV contractility. Conversely, our findings show that changes in BCG amplitude due to physiological fluctuations and pathological events may be more challenging to distinguish, and this may limit its use as a stand-alone marker.

While promising, these results should be considered in light of the limitations of this study. The model predictions are based on the variations of a single parameter, *ELS*. While bearing particular relevance for LV contractility, *ELS* is not the only model parameter affecting cardiac function. A sensitivity analysis could be conducted on the cardiovascular model to identify the model parameters that influence the BCG waveform the most, especially in relation to TEB and BCG amplitude. Furthermore, the values of the model parameters utilized for the simulations reported in this work are based on literature for human subjects, while the experimental waveform are obtained on swine. While swine are widely accepted as reliable pre-clinical models for cardiovascular diseases (44, 53), studies on human subjects should be conducted to evaluate the clinical applicability of the method proposed in this work. While the feasibility of BCG acquisitions based on an accelerometer placed under the head pillow has been successfully validated on critically-ill patients in the Surgical Intensive Care Unit of the University Hospital—MU Health Care (18), further studies are required to establish a relationship between the model-predicted BCG changes and actual deterioration in cardiovascular function. These are very important directions for future research which, however, go beyond the scope of this work. Here, we aimed at providing experimental evidence that mechanism-driven modeling can be used as a guide to interpret cardiovascular signals and, in future works, such approach could be used to study other disease states, including heart failure, valvular stenosis, sepsis, and atherosclerosis. Ultimately, mechanism-driven modeling could help leverage fundamental research in physiology to develop effective non-invasive methods for monitoring cardiac function in the clinics and at home.

## Data Availability Statement

The datasets presented in this article are not readily available because some of the data generated was in collaboration with industry partners that would require inspection and approval regarding the presence or absence of intellectual property issues. Requests to access the datasets should be directed to guidobonig@missouri.edu.

## Ethics Statement

The animal study was reviewed and approved by University of Missouri Animal Care and Use Committee.

## Author Contributions

All authors have contributed to reviewing and finalizing the draft. GG, CE, MZ, JI, DT, and LS have contributed to drafting the manuscript. GG, MZ, MS, MP, JK, LD, SA, CE have contributed to the BCG-sensing technique and the interpretation of the results. CE, MZ, JI, DT, CM, PT, SK, KS, and AA have contributed to the data acquisition in swine. GG, MZ, and LS have contributed to the implementation of the mathematical model and its numerical simulations. GG, CE, and MZ have contributed to all aspects of the project.

## Funding

This research was partially supported by NIH RO1 HL112998 (CE), Department of Defense Grant W81XWH1810178 (CE and MK), NIH R44 HL140649 (PR-L and CE), and funding from Cardiac RSK3 Inhibitors, LLC (CE). The authors also acknowledge support from the Center of Eldercare and Rehabilitation Technology and the University of Missouri. The funder was not involved in the study design, collection, analysis, interpretation of data, the writing of this article or the decision to submit it for publication.

## Conflict of Interest

GG would like to disclose that she received remuneration from Foresite Healthcare LLC for serving as a consultant. MS also discloses a conflict with Foresite Healthcare LLC outside the submitted work and patents licensed to Foresite Healthcare LLC. PR-L holds equity in REGENCOR. MK holds equity in Anchored RSK3 Inhibitors, LLC, and Cardiac RSK3 Inhibitors, LLC. These relationships are pursuant to University of Missouri's policy on outside activities. The remaining authors declare that the research was conducted in the absence of any commercial or financial relationships that could be construed as a potential conflict of interest.

## Publisher's Note

All claims expressed in this article are solely those of the authors and do not necessarily represent those of their affiliated organizations, or those of the publisher, the editors and the reviewers. Any product that may be evaluated in this article, or claim that may be made by its manufacturer, is not guaranteed or endorsed by the publisher.
